# Clinical agreement in the American Society of Anesthesiologists physical status classification

**DOI:** 10.1186/s13741-018-0094-7

**Published:** 2018-06-19

**Authors:** Kayla M. Knuf, Christopher V. Maani, Adrienne K. Cummings

**Affiliations:** Department of Anesthesia, San Antonio Military Medical Center, 3551 Rodger Brooke Dr, Fort Sam Houston, San Antonio, TX 78234 USA

**Keywords:** Variation between specialties, Anesthesiology/standards, Preoperative care, Risk assessment/classification

## Abstract

**Background:**

The American Society of Anesthesiologists physical status (ASA-PS) classification is not intended to predict risk, but increasing ASA-PS class has been associated with increased perioperative mortality. The ASA-PS class is being used by many institutions to identify patients that may require further workup or exams preoperatively. Studies regarding the ASA-PS classification system show significant variability in class assignment by anesthesiologists as well as providers of different specialties when provided with short clinical scenarios. Discrepancies in the ASA-PS accuracy have the potential to lead to unnecessary testing and cancelation of surgical procedures. Our study aimed to determine whether these differences in ASA-PS classification were present when actual patients were evaluated rather than previously published scenario-based studies.

**Methods:**

A retrospective chart review was completed for patients >/= 65 years of age undergoing elective total hip or total knee replacements. One hundred seventy-seven records were reviewed of which 101 records had the necessary data. The outcome measures noted were the ASA-PS classification assigned by the internal medicine clinic provider, the ASA-PS classification assigned by the Pre-Anesthesia Unit (PAU) clinic provider, and the ASA-PS classification assigned on the day of surgery (DOS) by the anesthesia provider conducting the anesthetic care.

**Results:**

A statistically significant difference was shown between the internal medicine and the PAU preoperative ASA-PS designation as well as between the internal medicine and DOS designation (McNemar *p* = 0.034 and *p* = 0.025). Low kappa values were obtained confirming the inter-observer variation in the application of the ASA-PS classification of patients by providers of different specialties [Kappa of 0.170 (− 0.001, 0.340) and 0.156 (− 0.015, 0.327)].

**Conclusions:**

There was disagreement in the ASA-PS class designation between two providers of different specialties when evaluating the same patients with access to full medical records. When the anesthesia-run PAU and the anesthesia assigned DOS ASA-PS class designations were evaluated, there was agreement. This agreement was seen between anesthesia providers regardless of education or training level. The difference in the application of the ASA-PS classification in our study appeared to be reflective of department membership and not reflective of the individual provider’s level of training.

## Background

As the concept of a single surgical procedure has transitioned to a comprehensive perioperative process, the outcomes of many major elective operations have improved. Care now focuses on a preoperative evaluation, early planning for discharge, and post-procedure rehabilitation (Donabedian 1966; Bader 2012). This integrated perioperative system promotes the combination of the three care phases: preoperative, intraoperative, and postoperative. As this transition of perioperative ideology continues, patients will benefit from multidisciplinary management for effective and efficient patient care (Adamina et al. 2011; Perioperative Surgical Home n.d.).

The preoperative component requires comprehensive preoperative evaluations. This has resulted in a change from a simple day of surgery evaluation to the establishment of standardized preoperative clinics. The purpose of these more thorough preoperative clinics is to allow for deliberate and careful clinical evaluation with additional investigation and optimization of medical conditions as indicated to promote better patient outcomes and reduce unnecessary medical expenses. Studies have linked the implementation of preoperative clinics with improved patient outcomes such as decreased in-hospital mortality and cost-reduction due to a decrease in day of surgery cancelations (Hoyt n.d.; Blitz et al. 2016; Whitlock et al. 2015). There are many types of preoperative clinics with multiple staffing models including providers from a variety of specialties and training levels (Johnson et al. 2014).

There are several components to a preoperative evaluation, including the American Society of Anesthesiologists Physical Status (ASA-PS) classification which was established in the 1940s and has since undergone multiple revisions. While not intended to predict risk, increasing ASA-PS class has been associated with increased perioperative mortality (Lemmens et al. 2008; Hopkins et al. 2016). The incidence of perioperative morbidity also rises with increasing ASA-PS class from 3.9% in an ASA 1 to 33.7% in an ASA 4 (Menke et al. 1993). As the perioperative system of care evolves, many institutions are attempting to maximize value via patient stratification, i.e. requiring only patients with higher ASA-PS classification scores to undergo formal preoperative evaluation and allowing those with lower ASA-PS classification scores to bypass preoperative clinics in an effort to streamline care. This has important implications as the provider who assigns the initial ASA-PS class stratifies the patient to either further preoperative evaluation or preoperative bypass. While the ASA-PS classification is one component of the preoperative evaluation, it has important ramifications in perioperative medicine as well as the practice of anesthesia. The classification affects surgical decision making, the anesthetic plan, and billing/reimbursement practices. Due to these consequences, it is important to have a consistent application of the ASA-PS classification system across providers, clinics, and specialties.

Studies regarding the ASA-PS classification system show significant variability in class assignment by anesthesiologists when provided with short clinical scenarios or hypothetical vignettes (Owens et al. 1978; Cuvillon et al. 2011; Mak et al. 2002; Riley et al. 2014). Variability is also seen in retrospective chart review comparing the ASA-PS class assigned at a preoperative clinic versus the ASA-PS class assigned in the operating room (Sankar et al. 2014). Inter-rater reliability is not the only issue with the ASA-PS class system, but intra-rater reliability which one would expect to show near perfect agreement has shown only moderate agreement in the pediatric cancer setting (Tollinche et al. 2018). Not only is there disagreement between anesthesia providers, but providers of different specialties also lack consistency. A recent study administered a survey of clinical scenarios to anesthesia providers, surgeons, and internists. In this study, providers of different specialties not only assigned an ASA-PS classification score less consistently, but they also had a tendency to underrate the class of the patients when compared to anesthesia providers given the same scenario (Curatolo et al. 2017; Eakin and Bader 2017).

When clinical scenarios are used to study the assignment of the ASA-PS classes, there are many limitations. Study participants are unable to ask for additional information or to extract and analyze applicable data from the medical record. Our study seeks to retrospectively assess the consistency of the ASA-PS class assignment between anesthesia providers and internists when evaluating patients undergoing total hip and total knee replacements at our institution during a 2-year period (Table 1). Due to variability in training and exposure to the ASA-PS classification system, our hypothesis predicted disagreement between the ASA-PS classes assigned by internal medicine and anesthesia providers on the same patient when both providers complete a history and physical exam with access to the entire medical record.

**Table 1:** Patient demographics

Average age	73.5
Average BMI	30.3
Male	51.40%
Female	48.50%
Hip	30.70%
Knee	69.30%

## Methods

**Fig. 1:**
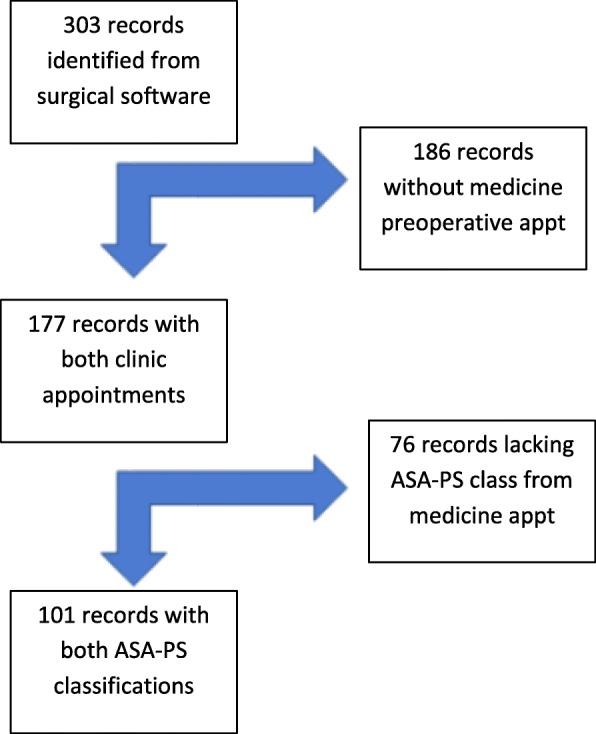
Consort diagram

After obtaining IRB approval, this single-center study was completed. Surgical scheduling software was queried for all patients >/= 65 years of age undergoing elective total hip or total knee replacements with surgical dates between 01 Jan 2015 and 31 Dec 2016 at a contemporary military treatment facility (MTF). A total of 303 patients were screened in the specified time period. These records were reviewed to eliminate emergent cases as well as to ensure that the patients had visited both the internal medicine preoperative clinic and the preoperative anesthetic unit (PAU). The resulting 177 records were reviewed of which 101 records were assigned an ASA-PS classification by both the medicine preoperative clinic and the PAU clinic (Table 2). These were included in the data analysis (Fig. 1).

**Table 2:** ASA-PS classification distribution by clinic

	Medicine	Anesthesia PAU	Anesthesia DOS
1	0	0	1
2	66	51	49
3	32	49	50
4	3	1	1

At our institution, surgeons and anesthesia providers can make referrals to the internal medicine preoperative clinic based on clinical judgment. There is no algorithm that establishes which patients would benefit from additional resources in the form of an internal medicine preoperative visit. There is a stratification process in which the surgeons can determine who completes a PAU clinic visit versus who can bypass the PAU. Bypass is reserved for ASA-PS 1 and 2 patients. These patients are contacted telephonically by the PAU to determine if there are any outstanding issues that may need to be addressed by a PAU visit. The surgeons can refer ASA-PS 1 and ASA-PS 2 to the PAU based on their preference or if the surgeon believes they would benefit from seeing an anesthesia provider prior to the day of surgery. The order in which these visits occur is variable as the appointments are booked by the patient. The ASA-PS classification used in this study was the ASA-PS classification assigned following the initial encounter by both the PAU and the internal medicine clinic (Table 3).

**Table 3:** ASA-PS class by PAU provider

Provider	Number	Percent
NP	27	27
PA	23	23
SRNA	4	4
Staff CRNA	39	39
Anes resident	5	5
Staff physician	2	2

For these records, the ASA classification from each visit as well as the day of surgery (DOS) ASA-PS class recorded by the anesthesia provider completing the case were collected. Supplemental data including age, BMI, gender, tobacco use, alcohol use, drug use, cardiac risk score, exercise tolerance (measured in metabolic equivalents), identified medical comorbidities, current medications, preoperative EKGs, additional preoperative cardiac study results, and preoperative pulmonary function test results were also collected (Table 4).

**Table 4:** ASA-PS class by DOS provider

Provider	Number	Percent
SRNA	11	11.1
Staff CRNA	26	26.3
Anes resident	40	40.4
Staff physician	22	22.2

The outcome measures noted were the ASA-PS classification assigned by the internal medicine clinic provider, the ASA-PS classification assigned by the PAU clinic provider, and the ASA-PS classification assigned on the DOS by the anesthesia provider. There is no formal training in assigning an ASA-PS classification in our internal medicine department. Training is provided to PAU providers that are not anesthesia trained, specifically the Nurse Practioners and the Physician Assistants that see patients in the clinic.

Data analysis software was used to perform the following analyses [SPSS v22.0 (IBM Corp. Released 2013. IBM SPSS Statistics for Windows, Version 22.0. Armonk, NY: IBM Corp)]. To assess the overall disagreement between the data sets, a McNemar test was completed with the following pairings: medicine and PAU, medicine and DOS, and PAU and DOS. To assess the overall agreement between the data sets, kappa statistics along with 95% confidence intervals were calculated for the aforementioned pairings (Table 5).

**Table 5:** McNemar and Kappa statistic

	Medicine versus PAU	Medicine versus DOS	DOS versus PAU
Kappa CI: (LB, UB)	0.170 (− 0.001, 0.340) *p* = 0.057	0.156 (− 0.015, 0.327) *p* = 0.079	0.863 (0.696, 1.030) *p* = 0.000
McNemar	6.769 (*p* = 0.034)	7.400 (*p* = 0.025)	0.143 (*p* = 0.705)

## Results

Three ASA-PS classifications documented by separate medical providers in reference to the same patient were obtained via retrospective chart review. The source of these ASA-PS classification sets were from the internal medicine preoperative appointment, the anesthesia PAU appointment, and the DOS anesthesia record. Medicine preoperative ASA-PS classifications were performed by resident physicians from the Department of Medicine with staff physician supervision. ASA-PS classifications from the PAU were performed by anesthesia providers and non-anesthesia providers with varying levels of experience, while those from the DOS were performed solely by anesthesia providers. The levels of experience included Physician Assistants (PAs) working in the PAU, Nurse Practitioners (NPs) working in the PAU, Student Registered Nurse Anesthetists (SRNAs), Certified Registered Nurse Anesthetists (CRNAs), Anesthesiology Residents, and Staff Anesthesiologists.

One record was excluded from the analysis, as it was designated an ASA-PS of 1 by the DOS anesthesia provider but as an ASA-PS of 2 by both the medicine and the PAU provider. Due to the fact that there were no other ASA-PS 1 designations in the data set, the McNemar test could not be performed. The McNemar test can be used only on paired nominal data; thus, the model could not be met as there was only 1 observed value of ASA-PS class 1.

When the ASA-PS class designation was compared between the internal medicine and the PAU preoperative assessment as well as between the internal medicine preoperative assessment and DOS designation, there was a statistically significant difference (McNemar *p* = 0.034 and *p* = 0.025, respectively). On further analysis of these groups, low kappa values were obtained further confirming the inter-observer variation in the application of the ASA-PS classification of patients by providers of different specialties [Kappa of 0.170 (− 0.001, 0.340) and 0.156 (− 0.015, 0.327), respectively].

Among the sets of ASA-PS classification from the PAU and the DOS, the low McNemar value demonstrates that the null hypothesis of marginal homogeneity cannot be rejected in respect to these two data sets indicating that these two sets of data are not in disagreement. Furthermore, the kappa value for these two sets of classifications was 0.863 (0.696, 1.030) indicating near perfect agreement between the two groups regarding the ASA-PS class assigned.

## Discussion

The goal of this study was to determine inter-rater reliability of the ASA-PS assignment between anesthesia and internal medicine providers in two preoperative clinics. We found disagreement in the designated ASA-PS classification between these two providers when evaluating the same patient with access to his or her full medical record. When the anesthesia-run PAU and the anesthesia assigned DOS ASA-PS class designations were evaluated, there was agreement. Interestingly, over half of the PAU evaluations in this study were completed by PAs or NPs from the department of anesthesia. These were non-anesthesia providers who were oriented and trained by licensed anesthesia providers. Approximately half of the DOS evaluations were completed by staff physicians and staff CRNAs while the other half were completed by trainees (with either direct or indirect supervision by a privileged anesthesia provider). There was agreement seen between anesthesia department staff regardless of education or training level. The difference in the application of the ASA-PS classification in our study appeared to be reflective of department membership and not reflective of the individual provider’s level of training.

The agreement in ASA-PS assignment seen in the anesthesia department at our institution regardless of training level suggests that the standard application of the classification system can be taught and learned. It also specifically implies that non-anesthesia providers could more predictably rate ASA-PS after education and brief training sponsored by the Anesthesia Department. This competency could be achieved independent of education or training level. Improving the inter-rater reliability between providers of different specialties will improve communication, preoperative risk stratification patient optimization, and perioperative care. To our knowledge, no study has looked at ASA-PS classification between providers of different specialties using a retrospective review of existing patient data. Prior studies utilized surveys of hypothetical clinical scenarios focusing on straightforward medical problems without clinical evaluation or correlation. These studies had a correct or designated ASA-PS class which was used to evaluate the accuracy of responders. While “correctness” can be determined in hypothetical, “static” clinical scenarios, it cannot always be determined in clinical situations with an actual patient. In “real-life” clinical situations that are often evolving or dynamic, it is the inter-rater reliability that is most useful in the preoperative management of patients.

While the ASA-PS class designation by the anesthesia provider on the day of surgery is the only ASA-PS class that matters in regard to billing and charting, there are potential clinical implications to non-anesthesia providers assigning an ASA-PS class early in the perioperative process. According to the American College of Cardiology/American Heart Association (ACC/AHA) Guidelines on Perioperative Cardiovascular Evaluation and Management of Patients Undergoing Noncardiac Surgery, assessment is made of a major adverse cardiac event (MACE) which leads to further workup or proceeding directly to surgery (Fleisher et al. 2014). While this was traditionally done with the Revised Cardiac Risk Index (RCRI), two new tools, the Gupta Myocardial Infarction or Cardiac Arrest (MICA) calculator as well as the National Surgical Quality Improvement Program (NSQIP) Surgical Risk Calculator, are mentioned in the guidelines. Both of these tools require the assignment of a ASA-PS class to produce the estimated perioperative risk of MACE. These risk tools are utilized by non-anesthesia providers and are a part of the perioperative cardiac assessment which determines which patients require further testing prior to noncardiac surgery.

Not only is accuracy of the ASA-PS class necessary to ensure the appropriate preoperative workup, but consistent ASA-PS classification also ensures accuracy of survival prediction models as well as quality comparisons among institutions (Skaga et al. 2007; Kuza et al. 2017). The ASA-PS class is also used by NSQIP to compare quality of care among hospitals. A recent study showed that the misclassification of the ASA-PS class significantly impacted the observed/expected mortality leading to skewed data in quality assessment between institutions (Helkin et al. 2017).

Our study had several limitations. While the retrospective nature of this study eliminated some of the shortcomings of prior studies, it introduced new limitations inherent to a retrospective study. Specifically, we were unable to collect full data sets as the ASA-PS class was not measured in a large subset of the patient population. Additionally, all data was collected retrospectively from the medical record; thus, if either provider did not take a full medical history and account for all medical comorbidities that in and of itself could explain the differences in the ASA-PS classification. Secondly, due to the inclusion criteria used, ASA-PS classes 1 and 5 were not represented in this study. While this is likely not clinically relevant, without full representation of all classes, we were unable to determine the applicability of the results to ASA classes 1 and 5. Thirdly, a large number of medical records were excluded due to insufficient data. Specifically, the most common reason for an incomplete data set was that the ASA-PS classification was missing from the medicine preoperative appointment. If these 76 records had been included, the results and significance of the study may have been different. Lastly, this retrospective study was completed at a military treatment facility (MTF) which had several implications. The patient population consisted solely of active duty military, retirees, and their dependents. These patients had increased access to care and decreased cost of care when compared with a civilian population. As a result, this population may have had an improved baseline health status when compared with a civilian population which may have resulted in less patient variability.

While this study was retrospective in nature and conducted at a MTF, we believe that the results are applicable to civilian facilities. The disagreement between providers’ use of the ASA-PS classification system as well as lack of uniformity in preoperative evaluations offers an opportunity for improving perioperative outcomes and patient safety. As comprehensive perioperative care continues to expand in a multidisciplinary fashion, preoperative evaluations form the cornerstone of patient stratification and resource allocation. If evaluations cannot be completed in an appropriate and consistent manner across perioperative providers, there is the potential for increased cost and decreased quality of care.

While research shows the inconsistencies that exist in the application of the ASA-PS classification system, further study is needed to determine how to solve this issue. It is difficult to ascertain the etiology of the inconsistency. Is it secondary to a lack of knowledge, or does it point to a deeper issue with the classification system we use? The next step would be to design an educational intervention that focuses on application of a consistent approach to the ASA-PS classification system. If this intervention results in improvement of inter-rater reliability between specialties, the likely explanation is a lack of knowledge/familiarity.

## Conclusions

In summary, there was a statistically significant difference in the application of the ASA-PS classification system between providers of the internal medicine department and the anesthesia department. In a clinical setting, the “right” ASA-PS classification is not nearly as important as reliable ASA-PS class designations between providers. The agreement between anesthesia providers of varying levels of training shows that consistent application is possible.

## Declarations

The view(s) expressed herein are those of the author(s) and do not reflect the official policy or position of Brooke Army Medical Center, the U.S. Army Medical Department, the U.S. Army Office of the Surgeon General, the Department of the Air Force, the Department of the Army, the Department of Defense, or the U.S. Government.

## Abbreviations

ACC/AHA: American College of Cardiology/American Heart Association Guidelines
ASA-PS: American Society of Anesthesiologists physical status
DOS: Day of Surgery
MACE: Major Adverse Cardiac Event
MICA: Myocardial Infarction or Cardiac Arrest
NSQIP: National Surgical Quality Improvement Program
PAU: Pre-Anesthesia Unit
RCRI: Revised Cardiac Risk Index
